# An integrated methodology for assessing ecosystem response to environmental stressors under changing climatic conditions in coastal wetlands

**DOI:** 10.1016/j.heliyon.2023.e21263

**Published:** 2023-10-20

**Authors:** Junghyung Ryu, Kam-biu Liu, Terrence A. McCloskey, Sang-Leen Yun

**Affiliations:** a Department of Oceanography, Division of Earth and Environmental System Sciences, Pukyong National University, 45 Yongso-ro, Nam-gu, Busan, 48513, Republic of Korea; b Department of Oceanography and Coastal Sciences, College of the Coast and Environment, Louisiana State University, Energy, Coast & Environment Building, Baton Rouge, LA, 70803, United States of America; c Independent Researcher, St. Margarets Village, Mile 32 Hummingbird Highway, Belize, Central America; d Department of Environmental Research, Korea Institute of Civil Engineering and Building Technology, 283 Goyang-daero, Gyeonggi-do, Ilsanseo-gu, 10223, Republic of Korea

**Keywords:** Global climate change, Environmental stressors, Integrated methodology, Grain size analysis, Geochemistry, X-ray fluorescence

## Abstract

Three cores were taken along the salinity gradient (n-s) in the coastal wetlands of Louisiana; an intermediate marsh, a brackish marsh, and a mangrove swamp. The cores display remarkable stratigraphic and chronologic correlations, representing six successive ecosystems and environments, namely: interdistributary bay, freshwater marsh/swamp, deltaic lake, freshwater marsh/swamp, intermediate marsh, and brackish/saline. Sedimentary, geochemical, and palynological data were used to reconstruct the paleoenvironments, including ambient environment and ecosystem types. Concentrations of Ba and Br, along with six elemental ratios (Ca/Rb, Zr/Rb, Ti/Rb, K/Ti, Mn/Rb, S/Rb), were employed to infer proxies for a range of environmental conditions (waterlogging, redox levels), depositional processes (fluvial vs marine or *in situ*), and sediment characteristics (grain size). Correlating the identification of environment types, inferred depositional processes, and the known history of the Mississippi delta cycle with the ecosystem reconstruction provides insight into ecosystem response to a variety of stresses, which information can be used to better understand and predict present and future responses to the ongoing stresses. Additionally, a simple elemental ratio (Zr/Rb) was used to produce a continuous (2 cm resolution) estimate of grain size along the length of the cores. The close correlation between the estimated grain size and measured samples shows that this ratio is a valid method for quickly assessing rough grain size, and is especially useful for identifying sedimentary inflection points.

## Teaser

**Synopsis**Utilizing multiproxy sedimentary and ecological data with precise chronology reveals coastal environmental changes with identifying ecosystem responses to various stressors.

## 1 Introduction

Global warming and the resultant rise in sea levels are driving critical levels of stress in most coastal regions [1,2]. With about half of the global population currently living in coastal areas [3], which function as infrastructure and transportation hubs as well as providing a plethora of ecological services, these regions are of great economic and societal importance on both national and global scales. In many locations, the most dominant deleterious effect is oceanic transgression, which by inundating low-lying areas, results in land loss. However, eustatic sea level rise is not the sole factor in determining the rate and extent of coastal retreat. A more important factor is relative sea level rise (RSLR), the sea level rise (SLR) relative to the adjacent land formed. Natural processes can drive the vertical accretion of coastal landforms, resulting in a lower RSLR than eustatic SLR rate, thereby slowing land loss. Although vertical accretion rates (VARs) are, of course, highly variable and controlled by a large number of factors, the most important controls are the characteristics of the local vegetation, and the ability of the vegetation to successfully maintain growth under conditions of stress [4]. Thus, understanding the vegetative dynamics at any location is key to determining the local RSLR rate, and consequently, predicting the rate of future land loss. A more complete understanding of the stress-vegetation response process can aid in developing strategies to retard coastal retreat.

Multi-proxy, sedimentary-based paleoenvironmental reconstructions can be extremely useful in elucidating vegetative responses to changing environmental conditions. Grain size and chemical analyses can be used to determine the provenance of the deposited materials, from which the dominant physical delivery processes can be inferred. In turn, this information can be used to identify the external forcing agents and the nature of the controlling stressor [5]. Combining this with a palynological analysis establishing the type, quantity, and biodiversity level of the contemporary vegetation gives a long-term history of vegetative response to a variety of stressors, which, of course, is of great utility in understanding present and future responses.

Our example sites are located in southeast Louisiana in coastal wetlands of the lower Mississippi River. The Louisiana coast has suffered a loss of ∼5000 km^2^ since 1930 [6] and has experienced a number of different environmental stresses over the late Holocene, marked by rapid and drastic shifts in ecosystem types. We aim to identify and understand the timing and nature of the various stresses, the resultant vegetative shifts, and the forces driving these changes. The investigation is based on the multiproxy analysis of sediment cores extracted from three distinct modern environments along the north-to-south geographic/salinity gradient; namely an intermediate marsh, a brackish marsh, and a mangrove swamp located on an island in a saline lake separated from the Gulf of Mexico by only a narrow sand barrier.

Sedimentary materials in coastal environments can vary greatly geochemically, mineralogically, by shape, size, texture, and provenance, can be produced *in situ*, delivered fluvially, or by marine processes, and can be preserved intact or undergo diagenesis. When combined with an accurate chronology, the sedimentary signatures of such materials provide important information regarding the existent environment at any point in time. Elemental ratios can be used to infer the biogenic, terrestrial, marine, minerogenic, and organic nature of sediments, while grain size data can inform concerning the provenance of materials and the mode of transportation. Palynological data describes the existent ecosystem, which can be used to infer the general ambient conditions. The use of multivariate statistical techniques derived from these data can identify such factors as origin and type of sediment, discharge levels, waterlogging, redox levels, and periods of marine intrusions, and allows for the identification of the various coastal stresses and their changing relative importance over time. Combining these processes results in a holistic methodology that can be applied over a wide environmental spectrum to identify contemporaneous stresses and the resultant vegetative response.

Sedimentary-based paleoecological studies of coastal wetlands typically rely on grain size analysis as a means of inferring depositional/environmental conditions. However, although highly informative, the required methodologies can be extremely time-consuming, as they require either sieving or the use of a particle laser analyzer and the use of multiple runs, which are then averaged through a statistical program. Additionally, both methods require pretreatment to remove organic material. The geochemical dataset provided by X-ray fluorescence (XRF) spectrometry facilitates a high-resolution geochemical profile and provides a rapid, non-destructive method for providing a continuous, though less informative, grain size profile [7]. Various elemental ratios have been used as proxies to interpret grain-size changes in sediment sequences in fluvial and coastal studies [8]. Particular elements such as Zr, Rb, and Ti tend to align with specific grain-size fractions during transportation by water or wind, with Zr being associated with sand and coarse silt, while Rb primarily occurs with silt and clay-sized fractions [8]. Here we present a novel empirical method whereby the mean grain size and textual definition can be estimated at every tested interval (every two cm through the length of the core in our case), based on a simple elemental ratio. Although this method does not provide sorting, skewness, kurtosis, and modal properties, it provides an extremely quick method for developing continuous sedimentary profiles, wherein all grain size changes are immediately recognizable and can be used to identify optimal depths for detailed (sieving, particle size) analyses.

## 2 Study site

### 2.1 Geological setting

The coastal wetlands of Louisiana contain over 57 000 km^2^ of marshland, transitioning from fresh marsh in the north to a vast expanse of brackish and saline marshes farther south [9]. All three study sites (LS, LL, BC) are located within Barataria Basin, ∼110 km long and 50 km wide [10], which contains multiple streams and estuarine lakes (Lake Salvador, Little Lake, Bay Champagne) with average depths between ∼1.5 and 3 m [10]. The basin is situated between the Bayou Lafourche distributaries to the west and natural levees of the Mississippi River to the east and receives multiple freshwater input from Bayou des Allemands, Bayou Perot, and Bayou Rigolettes in the north, Bayou Lafourche in the west, and through the Mississippi River from the east. Saline water enters from the Gulf of Mexico.

**Fig. 1:**
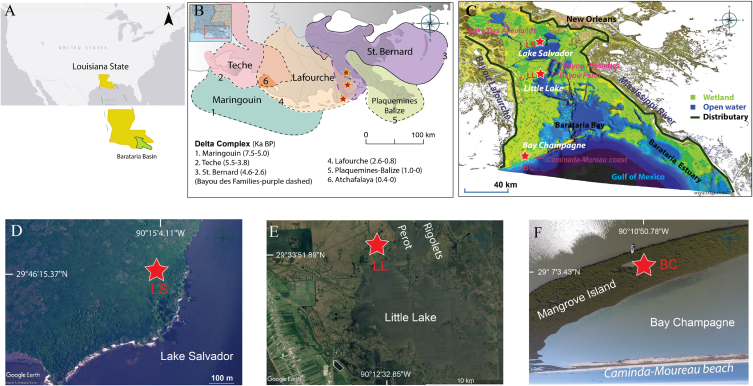
Map of study sites. (A) Map of the state of Louisiana (yellow) and Barataria Basin (green). (B) Holocene delta complexes of Mississippi delta. Study sites are marked with stars. (C) Map of Barataria Basin and locations of Des Allemands lake, Bayou Rigolettes, Bayou Perot, and Caminada-Moreau headland. (D, E, F) The proximity of the coring sites to Lake Salvador, Little Lake, Bay Champagne. Maps were produced using ArcGIS 10.3 and Google Earth.

Barataria Basin formed between 4000 and 3000 BP when the Mississippi subdeltas (the Bayou des Families and Bayou Barataria deltas) developed, achieving its present shape ∼2000 BP [11] (Fig. 1C). Multiple freshwater streams were blocked ∼300 BP as the natural levees along the Mississippi River developed, limiting the discharge of sediment and water into the basin [12]. The limited sediment supply and land subsidence, driven by the high compaction rates (>5 mm/yr), associated with the dewatering of Holocene deltaic sediments and rising sea levels have resulted in erosion and the northward migration of saline waters [13].

LS is an intermediate marsh located on the margin of Lake Salvador, ∼19 km southwest of New Orleans (Fig. 1D). Small patches of cypress trees and marsh plants around the lake are nourished by freshwater input from the Bayou des Allemands, while intense storm surges push saline water into the site. LL is a brackish marsh, located on the northern edge of Little Lake (Fig. 1E), which receives significant freshwater from Bayou Perot and Bayou Rigolettes, with occasional incursions of saline water from Barataria Bay. BC is a mangrove swamp located on an island in Bay Champagne on the Caminada-Moreau headland in the southernmost part of the Barataria estuary (Fig. 1F). Due to oceanic transgression, the ocean edge of Bay Champagne has retreated >2 km since the 1930s CE, [14].

### 2.2 Paleoenvironmental setting

Most (if not all) coastal wetland locations in Louisiana have experienced a succession of ecosystem changes during the late Holocene, driven by changing environmental conditions. The proximate causes of these changes are varied, ranging from local (anthropogenic activities), to regional (delta lobe switching), to global (sea level rise). Common ecosystem types are freshwater *Taxodium* swamp, freshwater marsh, lacustrine/subaqueous environments, intermediate marsh, brackish marsh, saline marsh, mangrove swamp, and interdistributary bay [15], with most locations having transitioned through several ecosystem types, generally in a pattern of increasing salinity. A prominent driver of these ecosystem alterations is the Mississippi River deltaic cycle, with freshwater environments, newly created marsh, and swamps occurring during delta progradation, and land subsidence and oceanic transgression occurring during the abandonment phase [16].

### 2.3 Paleoecological setting

Fossil pollen accurately reflects the composition of plant communities in the past and is the most commonly used tool to reconstruct paleoenvironments. With the examination of fossil pollen, researchers can discern and comprehend the profound shifts in plant assemblages, thus shedding light on the corresponding alterations in environmental conditions such as climate patterns and overall ecological dynamics. This provides invaluable insights into environmental transformations and the stresses that drive ecosystem changes. In this study, palynological data is used to infer the ambient ecosystem/plant assemblage during deltaic environmental shits. The changes in plant assemblages/vegetation type reflect changing environmental conditions in coastal wetlands. For example, the replacement of upland plants by aquatics indicates increased water depth at the location. The specific relationships between pollen records and vegetation pressures were adapted from previous publications [[1], [2], [3], [4]].

### 2.4 Sea level, subsidence, and tropical cyclones

The current rate of relative sea-level rise (RSLR) along the Gulf coast varies from 5 to 12 mm/yr [17], a result of a combination of eustatic SLR and land subsidence driven by a compaction rate of 0.05–0.25 mm/yr for unconsolidated sedimentary [18]. Anthropogenic activities (water and petroleum extraction, channel digging) are partially responsible for the high local variability in subsidence rates. These processes, further amplified by the battering of storms, have resulted in coastal erosion and the enlargement of wetland water bodies.

Tropical cyclones, including major hurricanes such as Betsy (1965), Camille (1969), Andrew (1992), Katrina (2005), Rita (2005), Gustav & Ike (2008), Laura & Delta (2020), and Ida (2021) have frequently struck Louisiana's coast. Such hurricanes generate large storm surges (>3 m), resulting in massive inland flooding and destruction of the existing vegetation. The deposited material often contains detectable chemical and textural signatures, which can be used to identify their occurrence in the sedimentary record [19,20].

### 2.5 Climate setting

During the Late Holocene, the climate in the Gulf of Mexico (GOM) experienced fluctuations, shifting between periods of wet and warm to cold and dry. During the wet and warm periods increased evaporation over the GOM resulted in enhanced precipitation over continental North America, with the opposite conditions prevailing during the cold and dry periods [21]. This cycle has caused significant temporal variability in the amount of freshwater flowing through the Mississippi River, subsequently affecting the geological and ecological conditions in the coastal wetlands.

## 3 Materials and methods

### 3.1 Core recovery

More than ten cores (LS, LS1, LS2, LL, LL1, LL2, LL3, BC, BC1, BC2, BC3, BC4) were extracted using a push and vibracorer from an intermediate (LS), brackish (LL), and mangrove swamp (BC) along a north-south transect of coastal Louisiana between September 2017 and June 2018 (Fig. 1D–F). The longest core (LS, LL, BC) from each site was selected for geological, geochemical, and ecological analyses. The average length of the three cores was 4.35 m. The cores were vertically transported to the Department of Oceanography & Coastal Sciences at Louisiana State University and stored in a refrigerated room at 4 °C.

### 3.2 Sedimentary analyses

The three cores were split longitudinally, described visually, using the Munsell color chart to describe coloration, and photographed [22]. Loss-on-ignition (LOI) was performed at a 1-cm interval down the center of each core, following the procedure of [23], quantifying the percentages of water, organic, carbonate contents, and residuals (mainly silicates). Based on the LOI results, a grain-size analysis was performed for all cores at 5–20 cm intervals, following conventional procedures [24]. Particles <2000 μm were wet-sieved, while particles >2000 μm were dry-sieved. Twelve sediment samples (∼1 g) collected from the BC core were pretreated to remove organics and then subjected to particle size analysis using a Beckmann-Coulter laser diffraction unit (Model LS 13 320) [25]. Mean grain size, sorting, skewness, kurtosis, and textural group were reported for all samples.

### 3.3 Geochronology

Geochronology was completed using accelerator mass spectrometry (AMS) and radiochemical analyses (activities of ^137^Cs and ^210^Pb). Seventeen plant debris samples and a shell were collected (SI1) and sent to International Chemical Analysis, INC in Miami, FL for AMS ^14^C dating. More than seventy samples were extracted at 1 cm internals from the top 1 m section of the cores for ^210^Pb (natural ^238^U series) and ^137^Cs (thermonuclear byproduct fallout) radiochemical analyses. A gamma(γ)-ray spectrometer (GRS) calculated the activities of ^137^Cs and ^210^Pb using conventional methods [26]. The results are reported in decays per minute per gram (dpm/g, 1 dpm = 60 Bq).

An age-depth model was developed using Clam v2.2, cc4 = “mixed.14C″ datasets [27] with a regional reservoir offset of 200 [28,29]. The maximum peaks of ^137^Cs fallout (1963 CE) are marked.

### 3.4 X-ray fluorescence

An Innov-X Delta Premium X-ray fluorescence (XRF) analyzer, calibrated with NIST standards 2710a and 2711a, was used to scan the length of all cores at 2-cm intervals [30], reporting the concentration (ppm) of 32 elements. Elemental concentrations and ratios were developed as proxies to infer the biogenic (Ca/Rb), terrestrial (Zr/Rb, Ti/Rb, K/Ti), marine (Ba), minerogenic (Mn/Rb, S/Rb), and organic (Br) source/environmental conditions for each sample, as detailed in Discussion.

R software packages, "ggplot2″ and "reshape2″ were used to test a correlation between the zirconium (Zr)/rubidium (Rb) ratio and mean grain size [31].

### 3.5 Palynology

Over two hundred thirty samples (1.8 cc each) were collected at 5-cm intervals for pollen analysis. The conventional chemical treatments involving 10 % hydrochloric acid (HCl), 10 % potassium hydroxide (KOH), 49 % hydrofluoric acid (HF), acetolysis solution, glacial acetic acid, and tertiary butanol alcohol (TBA) were performed with exotic Lycopodium tablets added to each sample to quantify pollen concentration (Faegri and Iversen, 1975). A minimum of 300 pollen grains were counted for each sample, using a light microscope with the aid of published pollen guides [[32], [33], [34]]. Details of pollen identification (e.g., *Spartina*) followed the methodology described in previous studies [11,35].

## 4 Results

### 4.1 Granulometry

**Fig. 2:**
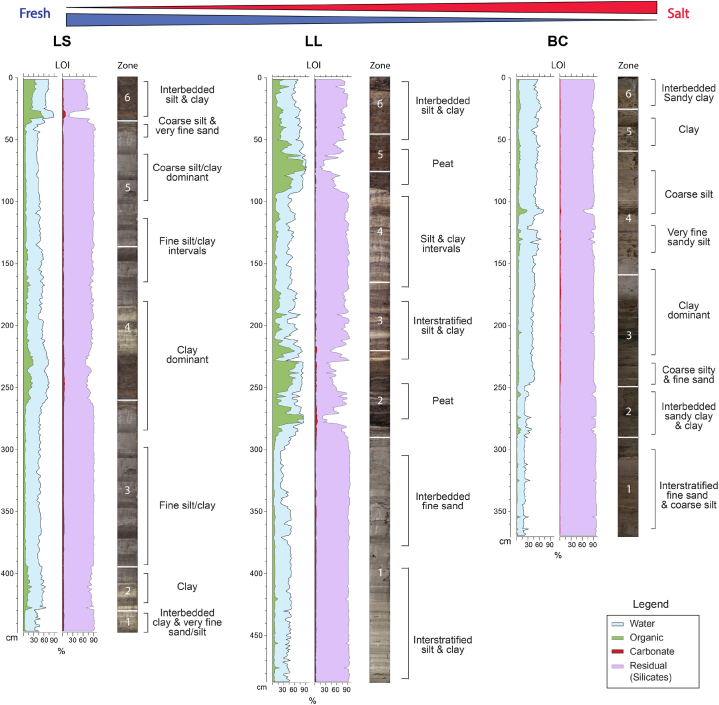
Core stratigraphy. Loss-on-ignition results and core photos are displayed with stratigraphic characteristics. Notice the close cross-core correlation.

All three cores display a general fining upward profile (Fig. 2), as reflected in the coloration with very coarse silt and very fine sand presenting as a light grey, and fine silt to clay as dark grey to black.

**Fig. 3:**
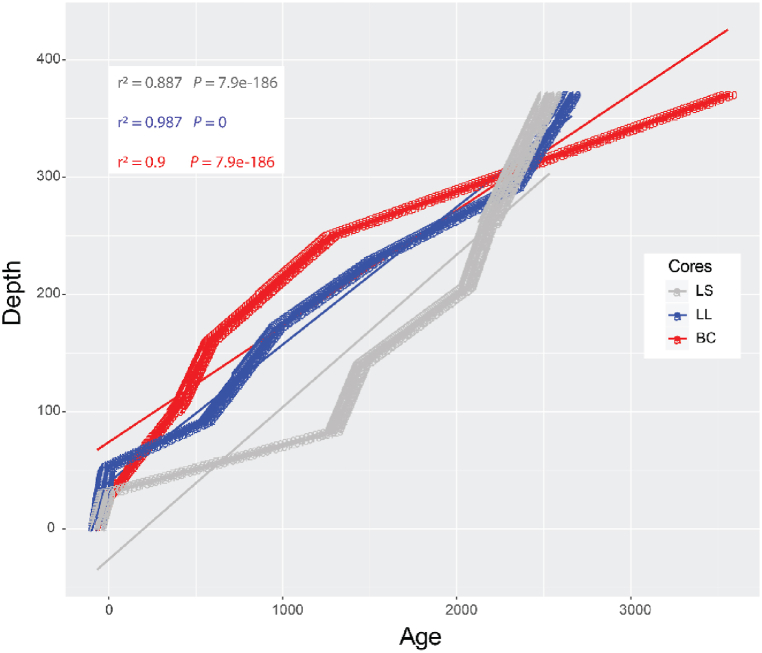
An age-depth regression plot for the three cores (LS, LL, and BC). All cores show significant geochronological correlations with *r*^2^ > 0.88 and *P* value < 0.01. Blue, grey, and red lines indicate LS, LL, and BC cores with trend lines, respectively.

The size distribution plots display unimodal, bimodal, trimodal, and polymodal groupings, with the texture classification ranging from sand to mud. The mean grain size of the sediments was highly variable, ranging from 72.6 to 11.43 μm in LS, 110.7 to 25.12 μm in LL, and 120.6 to 21.37 μm in BC (SI2–SI4), while overall stratigraphy is correlated across the cores with trimodal, poorly sorted very fine sandy mud to mud between 487 and 370 cm, unimodal, moderately sorted muddy sand between 370 and 300 cm, moderately sorted sand to sandy mud from 300 to 180 cm, and intermittent silt and clay between 255 and 220 cm (SI2–SI4). Bimodal to polymodal, poorly sorted very fine sand to silt presents between 180 and 60 cm, with sandy clay to clay between 60 and 0 cm. Average, clay, silt, and sand percentages are 11 %, 46 %, and 43 %, respectively. A linear correlation plot testing the stratigraphic correlations between the three cores resulted in significant correlations with *r*^2^ ≥ 0.88 and *p*-value <0.01, indicating that the three cores are highly correlated stratigraphically, and show no evidence of hiatuses or sedimentary disturbances (Fig. 3). The core chronology is detailed in section 4.3.

### 4.2 Sedimentary characteristics

Six Zones were identified in LS, LL, and BC, all marked by abrupt changes in the LOI profiles. The cores are stratigraphically correlated, with the transitions occurring at different chronological ages and depths, depending on distance from the coast at different times. The dominant texture types are fine sand to silt in Zone 1, sandy clay and silty clay in Zone 2, fine sand/silt to clay interstratification in Zone 3, silt and clay interval in Zone 4, silt and clay in Zone 5, and interbedded sandy clay to clay in Zone 6 (Fig. 2).

Across the cores, Zone 1 has 20–52 % water, 1–11 % organics, 1–4% carbonates, and 80–98 % residual (mainly silicates) contents. Zone 2 consists of 25–86 % water, significantly higher organics (3–73 %), 1–6% carbonates, and 21–95 % silicates. Zone 3 exhibits 27–77 % water, lower organics (2–44 %), 1–5% carbonates, and relatively high silicates (50–96 %). Zone 4 displays 36–78 % water, 4–36 % organics, 1–5% carbonates, and 46–94 % silicates. Zone 5, the interstratified silt and clay with embedded peaty clay consist of 36–92 % water, organics 4–82 %, 1–4% carbonates, and 14–94 % silicates. Zone 6 consists of 51–86 % water, 7–76 % organics, 1–8% carbonates, and 58–97 % silicates.

### 4.3 Chronology

**Fig. 4:**
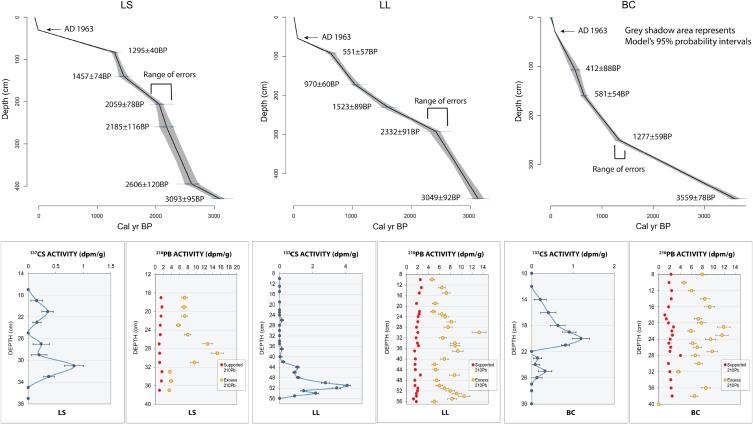
Age-depth curves and radiochemical trends. Age-depth model based on linear interpolation of radiocarbon ages (blue dots) and ^137^Cs peaks display core geochronologies with the range of errors for each sample (blue bars) shown as grey shadow areas, indicating 95 % probability intervals for Gaussian distribution. The results of ^210^Pb_xs_ and ^137^Cs analyses display ^137^Cs peaks and erratic ^210^Pb_xs_ activity. The geochronology was produced using Clam v2.2 software (Blaauw, 2010).

The calibrated radiocarbon dates range from 106 ± 108 to 3559 ± 78 cal yr BP (SI1). The radiocarbon dates indicate that the majority of the sediments were deposited during the active periods of the Bayou des Families (purple-dashed) and Lafourche (yellow) subdeltas (Fig. 1B). The radiochemical analyses yield ^137^Cs peaks (1963 CE) and the initiation of ^137^Cs activity (1954 CE) at depths of 31–34 cm in LS, 51–54 cm in LL, and 20–27 cm in BC (Fig. 4). In all three cores, ^210^Pb excess (^210^Pb_xs_) activities are erratic and lack a recognizable logarithmical decrease, likely due to the variability of sediment types, as the activity level decreases in coarser material, and increases in clays [15]. The age-depth linear interpolation curves were created using the radiocarbon dates and ^137^Cs activity curves, displaying five to six intervals of calibrated dates with a 95 % probability for Gaussian distribution displayed as a grey shadow [36]. The sediment accumulation rates (SARs) for the last 55 years (1963–2018), calculated from the ^137^Cs peaks, are from 0.92 to 0.36 cm/yr. Due to the erratic ^210^Pb_xs_ profiles, the ^210^Pb_xs_ SARs were not calculated.

### 4.4 X-ray fluorescence (XRF)

**Fig. 5:**
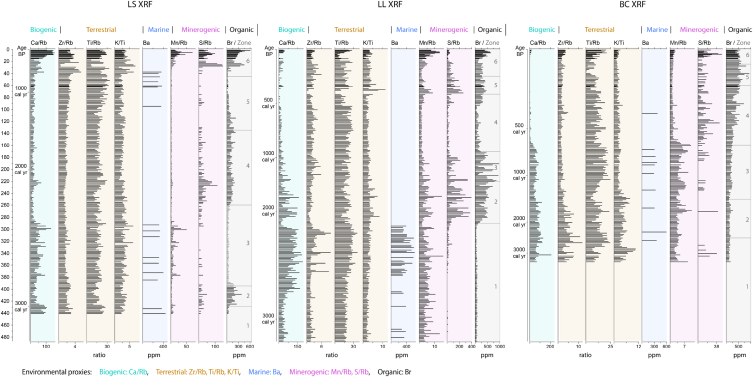
Geochemical results. The profiles of biogenic, terrestrial, marine, minerogenic, and organic proxies illustrate each zonation change.

Elemental ratios are employed to deduce biogenic (Ca/Rb), terrestrial (Zr/Rb, Ti/Rb, K/Ti), marine (Ba), minerogenic (Mn/Rb, S/Rb), and organic (Br) sources of deposited sediments (Fig. 5), as detailed in Discussion. Elemental concentrations vary temporarily and spatially as the environmental background conditions change over time and space. In general, the chemical signatures shift from high levels of terrestrial proxies during Zones 1–2, to increased organic sources (Br) during Zones 2–3, and to increasing minerogenic proxies under the lacustrine/subaqueous environments associated with oceanic transgression during Zones 3–6. Biogenic sources increase during Zone 1 and Zone 6. Ba elemental concentrations are inversely proportional to Br concentration, reaching maximum levels occurring during Zone 1.

### 4.5 Palynology

**Fig. 6:**
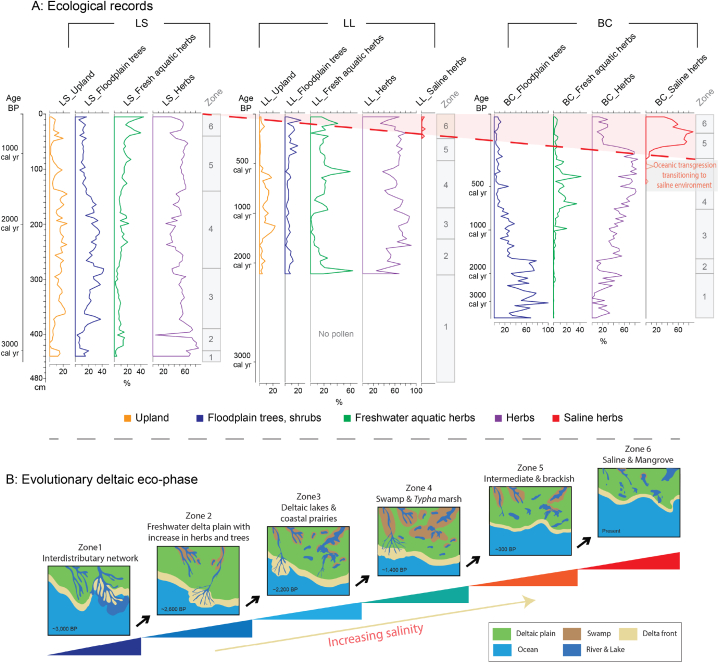
Pollen diagram and schematic representation of coastal evolution. A: Sum of pollen records of each core displaying pollen profiles (Upland, Floodplain trees & shrubs, Freshwater aquatic herbs, Herbs, Saline herbs) with calibrated radiocarbon dates. B: Schematic representation of coastal evolution displays ecosystem changes associated with deltaic growth. This figure was modified from Ryu et al., 2021.

Palynological analysis was used to determine the dominant ecosystem type for each Zone of each core, following the methodology of previously published articles [5,11,15]. The sums of pollen assemblages (upland, floodplain trees & shrubs, freshwater aquatic herbs, herbs, and saline herbs) represent temporally and spatially intercorrelated ecosystem changes (Fig. 6). Since ∼4000 BP, the sites, stratigraphically correlated with each other, display a common pattern of deltaic evolution, although time-transgressive, which varies depending on the distance from the coast; from interdistributary network (∼3000 BP in LS, ∼2300 BP in LL, ∼2000 BP in BC), to freshwater deltaic plain with an increase in freshwater herbs and trees between (∼2600 BP in LS, ∼1420 BP in LL, ∼1300 BP in BC), to deltaic lakes and coastal prairies (∼2200 BP in LS, ∼900 BP in LL, ∼700 BP in BC), to swamp or *typha* marsh (∼1400 BP in LS, ∼330 BP in LL), to intermediate or brackish (2000 CE in LS, 1972 CE in LL, ∼200 BP in BC), and to saline marsh (present in BC).

## 5 Discussion

### 5.1 Geochemical proxies

A variety of factors, such as water source (lake, river, ocean), sediment provenance, vegetation type, and microbial activities, affect the chemical composition of coastal sediments. These factors leave detectable physical and chemical signatures that enable the identification of the temporally varying coastal stressors [5,14,37]. Ba and Br concentrations and six elemental index ratios (Ca/Rb, Zr/Rb, Ti/Rb, K/Ti, Mn/Rb, S/Rb) were used to infer the source of materials and the associated sedimentary processes.

**Rb as a normalizing divisor:** Derived from the weathering of sedimentary rocks, Rubidium (Rb) is a common constituent in such rock-forming minerals as biotite, muscovite, feldspar, and illite [38,39]. Due to its great abundance as detrital minerals in sediments, Rb is extensively dispersed in natural environments, and can, thereby, be used as a divisor for normalizing other conservative elements [39].

**Ca/Rb:** Calcium (Ca) is present in large amounts in coastal sediments as a result of the decomposition of biogenic calcium carbonate (molluscan) or reworked coastal sediments [40,41]. The Ca/Rb ratio indicates a relative abundance of biogenic calcite, as nearly all Ca in coastal sediments is detrital carbonate derived from calcareous organisms. Ca can also (usually) exist as broken-up shells, foraminifera, etc.

**Zr/Rb:** Zirconium (Zr) is found in small quantities in minerals derived from metamorphic rocks, often in the form of zirconium (IV) silicate quartz, which is widely dispersed in natural siliciclastic sand and silt. Since Rb is associated with finer grains (clays), a high Zr/Rb ratio in coastal sediments usually indicates fluvially transported coarse-grained material [42,43].

**Ti/Rb:** Titanium (Ti) is an abundant element widely occurring in metamorphic and igneous rocks, commonly in the forms of TiO_2_, FeTiO_3_, CaTiSiO_5_. As such material is not naturally occurring in coastal Louisiana wetlands [44], a high Ti/Rb ratio indicates the presence of fluvially-delivered coarse-grained allochthonous sediment, such as laterites and bauxites, abundant in the upper Mississippi River basin [45].

**K/Ti:** Potassium (K) is an alkali metal, found in minerals derived from igneous rocks. Since K is associated with such fine-grained terrestrial clays as illite, potassium mica, and potassium feldspar [37], in estuarine environments high K/Ti values indicate fluvially-delivered small-grained minerals.

**Mn/Rb:** Manganese (Mn) is the 12th most abundant crustal element, occurring in such Mn-bearing minerals as pyrolusite (MnO_2_) and rhodochrosite (MnCO_3_) [46]. When Mn enters aquatic environments, the soluble manganese leaches downward [47]. Because of its high redox sensitivity, Mn concentrations increase under nutrient- and toxicant-rich conditions due to an increased level of *in situ* diagenetic activity [48]. Therefore, an elevated Mn/Rb ratio indicates anoxic conditions under elevated water tables and, possibly, mineral-rich material input [49].

**S/Rb:** Sulfur (S) is a highly reactive nonmetallic, element, insoluble in water, which plays a critical role in redox-biogeochemical cycles [50]. In wetland environments, organic matter accumulates in oxygen-depleted soils and sediments, creating anaerobic conditions. Under these conditions, certain microorganisms known as sulfate-reducing bacteria (SRB) use sulfate ions (SO_4_^2−^) as an electron acceptor in their metabolism. In environments where iron is present, SRB can couple sulfate reduction with iron reduction, leading to the formation of iron sulfides. This is particularly relevant in certain wetland soils and sediments where iron is abundant and interacts with sulfate-reducing bacteria [50]. Therefore, a high S/Rb ratio indicates a reducing condition.

**Ba:** Barium (Ba) is an alkaline earth mineral, chemically similar to calcium. Unlike other earth metals, Ba does not occur as a free element in nature. Abundant Ba, in the form of barium sulfate (BaSO_4_) and witherite (BaCO_3_), a widely dispersed oceanic mineral, can be found in coarse sediments [51], associated with a variety of depositional processes (biogenic, hydrothermal, and evaporation) [52]. In coastal environments high concentrations of Ba likely result from relict oceanic sediment trapped in an enclosed bay environment [53].

**Br:** Bromine (Br), a halogen, is widely distributed in the environment, usually occurring at low levels in soil and plants under normal conditions. Br is quite reactive, especially with organic compounds, because of its ability to initiate addition or substitution reactions. For this reason, Br concentrations are widely used as a proxy for biomass [49].

Using these elemental concentrations and ratios as proxies for biogenic, terrestrial, marine, minerogenic, and organic sources/environmental processes of sediments aids in inferring the existent hydrological and geological conditions (see Table 1).

**Table 1:** List of ratios and proxies.

Ratio/Element	Type of proxies	Proxies for
**Ca/Rb**	Biogenic	Biogenic calcite/Reworked coastal sediments
**Zr/Rb**	Terrestrial	Fluvially transported coarse-grained material
**Ti/Rb**	Terrestrial	Fluvially-delivered coarse-grained allochthonous sediment
**K/Ti**	Terrestrial	Fine-grained terrestrial clays
**Mn/Rb**	Minerogenic	Anoxic conditions under elevated water tables
**S/Rb**	Minerogenic	Reducing conditions
**Ba**	Marine	Relict oceanic sediment
**Br**	Organic	Biomass

### 5.2 Empirical grain-size analysis using X-ray fluorescence spectra

**Fig. 7:**
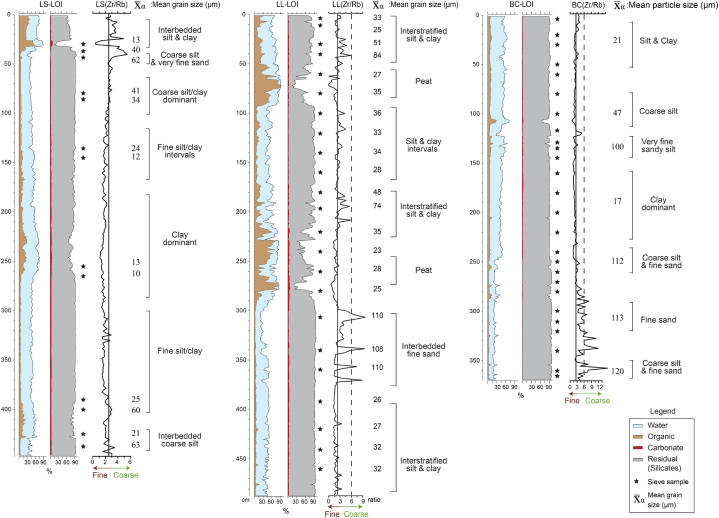
Lithology and Zr/Rb profiles of the LS, LL, and BC cores. The LOI data display water, organic, carbonate, and silicate percentages at 1-cm resolution. The Zr/Rb ratios are calculated at two-cm resolution. Stars indicate grain-size samples. Solid- and dashed-lines are the Zr/Rb ratio values of 2.5 and 6 respectively.

Given that the transport and deposition of materials in aqueous environments are closely linked to density and grain size, the grain size is extensively used to interpret geological and hydrological conditions, particularly with regard to assessing the energy level of the transporting fluid [54,55]. Thus, it is a particularly important tool in fluvial and/or coastal environments, as it can inform as to the occurrence of high energy events (e.g., storm surge, fluvial pulses). However, the standard laboratory methodology is laborious, time-consuming, and, in the case of laser particle analysis, expensive. To sidestep these drawbacks, we used a simple elemental ratio (Zr/Rb) to create an empirical estimation of sediment grain size at two cm resolution throughout the length of the cores [56,57]. A linear correlation plot testing the chronostratigraphic correlations between cores (LS, LL, BC) resulted in significant correlations with *r*^2^ ≥ 0.88 and *p*-value <0.01, indicating that the three cores are highly correlated stratigraphically with similar sedimentation patterns (Fig. 3). This estimation was then compared with the grain-size data as determined by conventional laboratory methods (sieving, laser particle analysis) (Fig. 7).

In general, silt-sand sections are marked by an elevated Zr/Rb ratio, while high percentages of organics and low percentages of silicates display decreased ratio values (Fig. 7). In order to differentiate fine versus coarse grains, solid- and dashed-lines were drawn at 2.5 and 6 ratio levels respectively. The sections containing clay to fine silt packages (478-400 cm, 250-200 cm, 100-70 cm in LL, 370-150 cm in LS, and 200-100 cm in BC) display low Zr/Rb ratios (<2.5), while the coarse silt to sand sections (360-300 cm in LL, and 370-290 cm in BC) are marked by elevated ratios (>6). In particular, episodic spikes in the Zr/Rb ratio (>6) are highly correlated with interbedded coarse silts to fine sands, reflecting particle size ranging between 108-120 μm, while Zr/Br ratios between 2.5 and 6 correlate with silt-dominant packages (∼40–100 μm) (SI2–SI4). Significantly low Zr/Rb values correlate with clay (high organics). Although the Zr/Rb ratio does not clearly distinguish between silt and sand, the grain-size pattern and Zr/Rb trend are remarkably similar. Hence, the Zr/Rb ratio can be used as a proxy for empirical grain size to determine coarse versus fine materials in deltaic environments. The Zr/Rb ratio is thus a quick way to estimate grain size and can be used to identify points of change, where more precise and detailed analysis can be performed.

### 5.3 Environment proxies

Environmental proxies are used to identify the source of sediments and/or the ambient environmental conditions at our sites, which are then used to infer the physical factors driving the ecological shifts at specific times.

**Zone 1 (**interdistributary network) displays a high level of terrestrial proxies, which can be associated with the progradation of the St. Bernard delta [16]. The core bottom represents an interdistributary bay (Fig. 6B) marked by elevated Ba concentrations. Then, in the early stage of delta progradation, massive amounts of terrestrial materials were progressively deposited over subaerial environments. Significant fluvial input over the bay environment likely redistributed/mixed sediments, evidenced by increased Zr/Rb, Ti/Rb, and Ca/Rb ratios.

**Zone 2** (freshwater deltaic plain with the increase in freshwater herbs and trees) is marked by high organics and elevated Br concentrations, indicating that marsh and swamps formed on the newly created deltaic plains resulted in the establishment of thriving terrestrial vegetation.

**Zone 3** (deltaic lakes and coastal prairies) displays a decrease in vegetative growth as a result of a delta lobe switching event that occurred between 3000 and 1000 years ago. This shift led to a reduced inflow of coarse sediment and freshwater, as evidenced by elevated K/Ti, Mn/Rb, and S/Rb ratios. These higher ratios indicate the presence of water-logged environments, such as potholes and lakes. The cause of this change was the Mississippi River altering its course to the southwest, leading to reduced hydrological efficiency in the southeast and the St. Bernard delta (old delta) (Fig. 6B). The rise in Ba levels indicates on-and-off oceanic transgressions due to the limited river flow to the southernmost site (BC).

**Zone 4** (swamps and *Typha* marsh) is marked by the reduced fluvial input over the old delta plain, which led to local subsidence and uneven topography, allowing the establishment of hydrophytes (*Typha*) in the intermediate environment. The elevated S/Rb ratio, synchronized or followed by Mn/Rb, suggests that water logging resulted in anoxic environments, with anaerobic environments following persistent inundation, marked by high sulfur levels in wet soil [58]. The newly developed Lafourche delta ∼1000 BP led to terrestrial input (Ti/Rb) with locally lush swamp ecosystems.

**Zone 5** (intermediate and brackish marshes) displays an increase in Br concentrations, which coupled with Mn/Rb and Sr/Rb ratios suggests that elevated levels of organic matter accumulated in oxygen-depleted soils and sediments during an oceanic transgression. During the transgressive phase of the Lafourche delta ∼300 BP, the significant reduction in fluvial input led to the incursion of saline water, while the concurrent land subsidence led to the creation of sunken grounds, marked by minerogenic processes. This resulted in the establishment of intermediate and brackish marshes along the coast. During this period, the northern site (LS) experienced a consistent and steady inflow of river water, indicated by the presence of elevated levels of terrestrial indicators, implying a stable freshwater environment.

**Zone 6** (saline and mangrove) is characterized by an increase in both mineral and biological proxies. During this time, the inactive delta lobe was retreating, and oceanic transgression inundated low-lying areas. The lack of oxygen caused the organic soil to degrade into anaerobic conditions, as identified by higher S/Rb ratios. Elevated Ca and Mn/Rb ratios indicate the increased occurrence of detrital carbonate under water-logged conditions, implying increased regional salinity due to oceanic transgression resulting from RSLR and storm surges. The identical transitions (increased salinity) occur in all three cores, although in a time-transitive manner, based on distance from the coast.

### 5.4 Ecosystem changes as correlated to contemporary stresses

We present the use of environmental proxies, which can be used to infer background environmental conditions (stresses). Due to the precise chronology, the changing conditions between Zones can be paired with the altered vegetation as determined by the pollen analysis, thus quantifying vegetative response to specific environment stresses. In general, chemical signatures shift from elevated levels of terrestrial proxies during the deltaic progradation (Zones 1–2), to an increase in Br concentration during the freshwater marsh/swamp developments (Zones 2–5), and to increasing minerogenic sources under the lacustrine/subaqueous environments associated with delta lobe switching and oceanic transgression (Zones 3–6). In Zone 1, the increase in Ba and the Ca/Rb ratio occurs during the early stages of delta progradation in the interdistributary network, a hydrologically unstable condition, causing the reworking/redistributing of sediments. The increases in the Zr/Rb and Ti/Rb ratios indicate the input of terrestrial materials and intense fluvial discharge during delta developments (St. Bernard and Lafourche: Zones 1 and 4). Elevated Br concentration occurs during periods of deltaic stability, and is a marker for the formation of marshes and swamps, and diverse ecosystems (Zone 2–3). S/Rb and Mn/Rb ratios reflect the retreat of the inactive delta lobe and an oceanic transgression to inundating low-lying areas. The anaerobic degradation of organic soil is identified by higher S/Rb levels. A notable rise in Ca/Rb and minerogenic proxies (Mn/Rb, S/Rb) indicates saline water inundation and sediment redistribution due to coastal flooding (e.g., storm surge).

### 5.5 Limitations

As with all paleoenvironmental studies, the lack of precise, accurate data and the possibility of unnoticed disturbances create limitations. This study focuses on a specific timeframe (the last 4000 years), which may not capture longer-term environmental changes or trends that occurred before this period. Extremely short-term deviations in the vegetative history may be missed. The accuracy and comprehensiveness of the environmental reconstruction depends on the availability and quality of historical data and proxies. Gaps or limitations in geological data sources could impact the study's conclusions. The study is focused on three specific locations, which possibly have eccentric histories that do not match surrounding areas. Therefore, our findings and the availability of the stream-lined grain size estimation may be specific to the coastal sites under investigation and may not be representative of deltaic coasts in other environments. Therefore, the use of multiproxy data and streamlined grain size estimation as tools to identify background parameters controlling coastal ecosystem changes assumes the reliability and accuracy of these methods.

## 6 Conclusion

Multiproxy sedimentary data and a tight chronology provide a means of identifying general environmental conditions at any time in the past. Changing environmental conditions (i.e., stress) can then be matched with ecosystem changes. The accurate identification of vegetative responses to specific stresses is extremely useful information and can be used to inform concerning present and future responses to environmental change. In this study, we reconstruct the environmental history of a coastal site over the last 4000 years, highlighting our view that the dominant external driver forcing ecosystem changes has varied over time.

Environmental proxies, used to infer background environmental conditions shifted from elevated levels of terrestrial proxies during the deltaic progradation (Zones 1–2), to an increase in Br concentration during the freshwater marsh/swamp developments (Zones 2–5), and to increasing minerogenic sources under the lacustrine/subaqueous environments associated with delta lobe switching and oceanic transgression (Zones 3–6).

The relative importance of the different environmental parameters varied both temporally and spatially. During the early stages of delta progradation in the interdistributary network, a hydrologically unstable condition caused the reworking/redistributing of sediments. The terrestrial influx and intense fluvial discharge during delta developments intensified wetland developments. An increase in organic accumulation occurred during periods of deltaic stability, constructing marshes and swamps with diverse ecosystems. During the inactive period of the delta lobe cycle, an oceanic transgression inundated low-lying areas, leading to the anaerobic degradation of organic soil. Accelerated coastal retreat caused saline water inundation and redistribution of sediments as a result of coastal flooding (e.g., storm surge).

Multiproxy analyses are an essential step in untangling the ecosystem successions on deltaic coasts. The stream-lined grain size estimation presented here can be a useful tool for identifying the relative importance of background parameters controlling coastal ecosystem changes.

## Data availability statement

Data will be made available on request.

## CRediT authorship contribution statement

**Junghyung Ryu:** Conceptualization, Data curation, Formal analysis, Investigation, Methodology, Resources, Software, Visualization, Writing – original draft, Writing – review & editing. **Kam-biu Liu:** Conceptualization, Data curation, Funding acquisition, Project administration, Supervision, Validation, Writing – review & editing. **Terrence A. McCloskey:** Investigation, Methodology, Writing – review & editing. **Sang-Leen Yun:** Visualization, Writing – review & editing.

## Declaration of competing interest

The authors declare that they have no known competing financial interests or personal relationships that could have appeared to influence the work reported in this paper.
