# Supplementary material for: An integrated methodology for assessing ecosystem response to environmental stressors under changing climatic conditions in coastal wetlands
Source: Heliyon. 2023 Oct 20;9(11):e21263. doi: 10.1016/j.heliyon.2023.e21263 (PMC10618510; doi:10.1016/j.heliyon.2023.e21263)
Supplement: Multimedia component 1 [file mmc1.docx]

**An Integrated Methodology for Assessing Vegetative Response to Environmental Stressors under Changing Climatic Conditions in Coastal Wetlands**

Junghyung Ryu^*^, Kam-biu Liu, Terrence A. McCloskey

*Corresponding author. Email: jhrainlv7@gmail.com

**This PDF file includes:**

Tables. SI1 to SI4

**SI1**. The complete radiocarbon data including possible date ranges for each sample and their probability percentages.

| **Sample ID** | **Sample** | **Depth (cm)** | **C14_age (yr)** | **Error (yr)** | **Cal_age (min)** | **Cal_age (median)** | **Cal_age (max)** | **Probability (%)** | **Cal_age**  **(final)** |
| --- | --- | --- | --- | --- | --- | --- | --- | --- | --- |
| LS1  19P/0721 | Plant debris | 83 | 1370 | 30 | 1264 | 1299.5 | 1335 | 95 | 1295±40 |
| LS2  18P/1046 | Plant debris | 140 | 1550 | 30 | 1379 | 1452.5 | 1526 | 95 | 1457±74 |
| LS3  19P/0722 | Plant debris | 206 | 2090 | 30 | 1993 | 2067 | 2141 | 95 | 2059±78 |
| LS4  18P/1047 | Plant debris | 260 | 2150 | 30 | 2010 | 2014.5 | 2019 | 0.9 | 2185±116 |
|  |  |  |  |  | 2040 | 2111.5 | 2183 | 64.1 |  |
|  |  |  |  |  | 2236 | 2270 | 2304 | 29.9 |  |
| LS5  19P/0723 | Plant debris | 395 | 2520 | 30 | 2491 | 2546.5 | 2602 | 50.2 | 2606±120 |
| LS6  18P/1048 | Plant debris | 430 | 2940 | 30 | 2993 | 3086.5 | 3180 | 94.4 | 3093±95 |
|  |  |  |  |  | 3201 | 3203 | 3205 | 0.6 |  |
| LL1  18P/1079 | Plant debris | 92 | 740 | 30 | 586 | 606 | 626 | 18 | 551±57 |
|  |  |  |  |  | 504 | 535 | 566 | 76.8 |  |
| LL2  18O/1140 | Plant debris | 173 | 1240 | 30 | 1018 | 1032.5 | 1047 | 11.3 | 970±60 |
|  |  |  |  |  | 921 | 967 | 1013 | 83.5 |  |
| LL3  18O/1141 | Plant debris | 230 | 1800 | 30 | 1471 | 1536 | 1601 | 80.2 | 1523±89 |
|  |  |  |  |  | 1419 | 1442.5 | 1466 | 14.6 |  |
| LL4  18P/1080 | Plant debris | 292 | 2480 | 30 | 2293 | 2358.5 | 2424 | 93.1 | 2332±91 |
|  |  |  |  |  | 2206 | 2216.5 | 2227 | 1.9 |  |
| LL5  18S/1081 | Shell | 464 | 3270 | 30 | 2955 | 3053 | 3151 | 94.9 | 3049±92 |
|  |  |  |  | R offset of 200 |  |  |  |  |  |
| BC1  17W/1136 | Plant debris | 53 | 40 | 30 | 32 | 57.5 | 83 | 56.2 | 106±108 |
|  |  |  |  |  | 97 | 102.5 | 108 | 3.3 |  |
|  |  |  |  |  | 111 | 124 | 137 | 15.1 |  |
|  |  |  |  |  | 223 | 239 | 255 | 20.5 |  |
| BC2  17W/1137 | Plant debris | 116 | 310 | 30 | 302 | 321.5 | 341 | 23 | 385±80 |
|  |  |  |  |  | 347 | 404.5 | 462 | 71.9 |  |
| BC3  17P/1138 | Plant debris | 169 | 560 | 30 | 523 | 543.5 | 564 | 45.2 | 582±56 |
|  |  |  |  |  | 589 | 615 | 641 | 49.7 |  |
| BC4  18O/0809 | Plant debris | 107 | 360 | 30 | 316 | 357.5 | 399 | 46 | 412±88 |
|  |  |  |  |  | 402 | 404 | 406 | 1.1 |  |
|  |  |  |  |  | 422 | 460 | 498 | 47.8 |  |
| BC5  18O/0637 | Plant debris | 160 | 570 | 30 | 523 | 543.5 | 564 | 45.2 | 581±54 |
|  |  |  |  |  | 589 | 615 | 641 | 49.7 |  |
| BC6  18O/0810 | Plant debris | 250 | 1350 | 30 | 1186 | 1195 | 1204 | 6.6 | 1277±59 |
| BC7 18O/0811 | Plant debris | 370 | 3330 | 30 | 1239 | 1275 | 1311 | 88.3 | 3559±78 |
|  |  |  |  |  | 3479 | 3557.5 | 3636 | 95 |  |

# SI2. LS granulometry: grain size distribution and statistic results.

|  | **SAMPLE STATISTICS** | | |  |  |  |  |  |  |  |  |  |  |  |
| --- | --- | --- | --- | --- | --- | --- | --- | --- | --- | --- | --- | --- | --- | --- |
|  |  | **30** | **40** | **45** | **80** | **85** | **135** | **145** | **255** | **265** | **390** | **400** | **425** | **435** |
|  | SAMPLE TYPE: | Bimodal, Poorly Sorted | Trimodal, Poorly Sorted | Trimodal, Very Poorly Sorted | Polymodal, Poorly Sorted | Trimodal, Very Poorly Sorted | Trimodal, Poorly Sorted | Polymodal, Very Poorly Sorted | Bimodal, Poorly Sorted | Bimodal, Poorly Sorted | Trimodal, Very Poorly Sorted | Trimodal, Moderately Sorted | Trimodal, Very Poorly Sorted | Bimodal, Poorly Sorted |
|  | TEXTURAL GROUP: | Sandy Mud | Muddy Sand | Muddy Sand | Sandy Mud | Muddy Sand | Muddy Sand | Sandy Mud | Mud | Sandy Mud | Muddy Sand | Muddy Sand | Sandy Mud | Sandy Mud |
|  | SEDIMENT NAME: | Very Fine Sandy Mud | Fine Silty Very Fine Sand | Fine Silty Very Fine Sand | Very Fine Sandy Fine Silt | Fine Silty Very Fine Sand | Fine Silty Very Fine Sand | Very Fine Sandy Fine Silt | Mud | Very Fine Sandy Mud | Fine Silty Very Fine Sand | Fine Silty Very Fine Sand | Very Fine Sandy Mud | Very Fine Sandy Mud |
| METHOD OF | MEAN | 11.43 | 72.60 | 61.17 | 41.27 | 62.78 | 61.77 | 23.99 | 12.73 | 11.43 | 61.07 | 83.62 | 32.85 | 13.14 |
| MOMENTS | SORTING | 26.61 | 43.41 | 43.56 | 44.21 | 42.11 | 43.16 | 39.69 | 31.32 | 26.61 | 40.17 | 39.44 | 35.43 | 29.12 |
| Arithmetic (mm) | SKEWNESS | 3.130 | -0.149 | 0.104 | 0.756 | 0.053 | 0.094 | 1.759 | 3.064 | 3.130 | 0.051 | -0.345 | 0.715 | 3.051 |
|  | KURTOSIS | 12.26 | 2.001 | 2.002 | 2.280 | 2.144 | 2.030 | 4.669 | 10.98 | 12.26 | 2.354 | 2.310 | 2.457 | 11.33 |
| METHOD OF | MEAN | 3.018 | 47.24 | 33.02 | 15.31 | 35.60 | 34.78 | 6.756 | 3.086 | 3.018 | 34.22 | 64.04 | 11.62 | 4.048 |
| MOMENTS | SORTING | 3.871 | 3.362 | 4.259 | 5.065 | 4.116 | 3.986 | 4.669 | 3.924 | 3.871 | 4.306 | 2.698 | 5.268 | 3.679 |
| Geometric (mm) | SKEWNESS | 1.354 | -1.363 | -1.016 | -0.104 | -1.191 | -1.014 | 0.673 | 1.432 | 1.354 | -1.269 | -2.296 | -0.064 | 1.095 |
|  | KURTOSIS | 4.037 | 3.481 | 2.599 | 1.554 | 3.025 | 2.595 | 2.387 | 4.420 | 4.037 | 3.170 | 7.764 | 1.484 | 3.918 |
| METHOD OF | MEAN | 8.372 | 4.404 | 4.921 | 6.029 | 4.812 | 4.846 | 7.210 | 8.340 | 8.372 | 4.869 | 3.965 | 6.427 | 7.949 |
| MOMENTS | SORTING | 1.953 | 1.749 | 2.090 | 2.340 | 2.041 | 1.995 | 2.223 | 1.972 | 1.953 | 2.107 | 1.432 | 2.397 | 1.879 |
| Logarithmic (f) | SKEWNESS | -1.354 | 1.363 | 1.016 | 0.104 | 1.191 | 1.014 | -0.673 | -1.432 | -1.354 | 1.269 | 2.296 | 0.064 | -1.095 |
|  | KURTOSIS | 4.037 | 3.481 | 2.599 | 1.554 | 3.025 | 2.595 | 2.387 | 4.420 | 4.037 | 3.170 | 7.764 | 1.484 | 3.918 |
| FOLK AND | MEAN | 1.893 | 36.06 | 35.22 | 12.56 | 35.30 | 35.29 | 7.334 | 1.895 | 1.893 | 35.06 | 82.51 | 7.484 | 3.166 |
| WARD METHOD | SORTING | 2.830 | 3.581 | 4.518 | 3.947 | 4.510 | 3.589 | 5.741 | 3.096 | 2.830 | 4.510 | 1.938 | 5.237 | 2.829 |
| (mm) | SKEWNESS | 0.946 | -0.587 | -0.654 | 0.629 | -0.656 | -0.587 | 0.279 | 0.947 | 0.946 | -0.660 | 0.116 | 0.220 | -0.345 |
|  | KURTOSIS | 1.068 | 2.003 | 0.770 | 0.768 | 0.774 | 0.526 | 1.238 | 1.222 | 1.068 | 0.779 | 2.000 | 0.671 | 1.076 |
| FOLK AND | MEAN | 9.045 | 4.794 | 4.828 | 6.315 | 4.824 | 4.824 | 7.091 | 9.044 | 9.045 | 4.834 | 3.599 | 7.062 | 8.303 |
| WARD METHOD | SORTING | 1.501 | 1.840 | 2.176 | 1.981 | 2.173 | 1.843 | 2.521 | 1.631 | 1.501 | 2.173 | 0.954 | 2.389 | 1.500 |
| (f) | SKEWNESS | -0.946 | 0.587 | 0.654 | -0.629 | 0.656 | 0.587 | -0.279 | -0.947 | -0.946 | 0.660 | -0.116 | -0.220 | 0.345 |
|  | KURTOSIS | 1.068 | 2.003 | 0.770 | 0.768 | 0.774 | 0.526 | 1.238 | 1.222 | 1.068 | 0.779 | 2.000 | 0.671 | 1.076 |
| FOLK AND | MEAN: | Clay | Very Coarse Silt | Very Coarse Silt | Medium Silt | Very Coarse Silt | Very Coarse Silt | Fine Silt | Clay | Clay | Very Coarse Silt | Very Fine Sand | Fine Silt | Very Fine Silt |
| WARD METHOD | SORTING: | Poorly Sorted | Poorly Sorted | Very Poorly Sorted | Poorly Sorted | Very Poorly Sorted | Poorly Sorted | Very Poorly Sorted | Poorly Sorted | Poorly Sorted | Very Poorly Sorted | Moderately Sorted | Very Poorly Sorted | Poorly Sorted |
| (Description) | SKEWNESS: | Very Coarse Skewed | Very Fine Skewed | Very Fine Skewed | Very Coarse Skewed | Very Fine Skewed | Very Fine Skewed | Coarse Skewed | Very Coarse Skewed | Very Coarse Skewed | Very Fine Skewed | Coarse Skewed | Coarse Skewed | Very Fine Skewed |
|  | KURTOSIS: | Mesokurtic | Very Leptokurtic | Platykurtic | Platykurtic | Platykurtic | Very Platykurtic | Leptokurtic | Leptokurtic | Mesokurtic | Platykurtic | Very Leptokurtic | Platykurtic | Mesokurtic |

# SI3. LL granulometry: grain size distribution and statistic results.

|  | **SAMPLE STATISTICS** | | |  |  |  |  |  |  |  |  |  |  |
| --- | --- | --- | --- | --- | --- | --- | --- | --- | --- | --- | --- | --- | --- |
|  |  | **5** | **11** | **30** | **40** | **60** | **80** | **100** | **120** | **140** | **160** | **180** | **195** |
|  | SAMPLE TYPE: | Polymodal, Poorly Sorted | Polymodal, Poorly Sorted | Trimodal, Poorly Sorted | Trimodal, Poorly Sorted | Polymodal, Poorly Sorted | Polymodal, Poorly Sorted | Polymodal, Poorly Sorted | Polymodal, Poorly Sorted | Polymodal, Poorly Sorted | Bimodal, Poorly Sorted | Trimodal, Poorly Sorted | Trimodal, Poorly Sorted |
|  | TEXTURAL GROUP: | Sandy Mud | Sandy Mud | Sandy Mud | Muddy Sand | Sandy Mud | Sandy Mud | Sandy Mud | Sandy Mud | Sandy Mud | Sandy Mud | Sandy Mud | Muddy Sand |
|  | SEDIMENT NAME: | Very Fine Sandy Coarse Silt | Very Fine Sandy Coarse Silt | Fine Sandy Coarse Silt | Coarse Silty Fine Sand | Very Fine Sandy Coarse Silt | Very Fine Sandy Coarse Silt | Very Fine Sandy Coarse Silt | Very Fine Sandy Coarse Silt | Very Fine Sandy Coarse Silt | Very Fine Sandy Coarse Silt | Very Fine Sandy Coarse Silt | Coarse Silty Fine Sand |
| METHOD OF | MEAN | 33.90 | 25.56 | 51.64 | 84.98 | 27.43 | 35.10 | 36.50 | 33.73 | 34.97 | 28.67 | 48.48 | 74.68 |
| MOMENTS | SORTING | 40.81 | 34.64 | 49.95 | 51.57 | 34.28 | 38.92 | 40.71 | 38.86 | 38.58 | 35.18 | 47.47 | 52.13 |
| Arithmetic (mm) | SKEWNESS | 1.597 | 2.203 | 0.728 | -0.491 | 1.943 | 1.570 | 1.450 | 1.603 | 1.544 | 2.103 | 0.863 | -0.104 |
|  | KURTOSIS | 3.961 | 6.569 | 1.708 | 1.388 | 5.728 | 4.027 | 3.592 | 4.155 | 4.012 | 6.036 | 2.013 | 1.185 |
| METHOD OF | MEAN | 18.28 | 13.85 | 29.54 | 58.33 | 14.98 | 21.26 | 21.24 | 19.52 | 20.95 | 17.65 | 28.17 | 48.99 |
| MOMENTS | SORTING | 3.093 | 2.870 | 3.060 | 2.843 | 2.954 | 2.660 | 2.804 | 2.783 | 2.710 | 2.488 | 2.999 | 2.879 |
| Geometric (mm) | SKEWNESS | -0.036 | 0.373 | -0.105 | -0.978 | 0.227 | 0.285 | 0.231 | 0.317 | 0.261 | 0.614 | -0.132 | -0.631 |
|  | KURTOSIS | 3.246 | 3.281 | 2.543 | 2.846 | 2.893 | 3.010 | 2.777 | 2.740 | 2.800 | 3.485 | 2.743 | 2.323 |
| METHOD OF | MEAN | 5.774 | 6.174 | 5.081 | 4.100 | 6.061 | 5.556 | 5.557 | 5.679 | 5.577 | 5.824 | 5.150 | 4.351 |
| MOMENTS | SORTING | 1.629 | 1.521 | 1.613 | 1.507 | 1.563 | 1.411 | 1.488 | 1.477 | 1.438 | 1.315 | 1.584 | 1.526 |
| Logarithmic (f) | SKEWNESS | 0.036 | -0.373 | 0.105 | 0.978 | -0.227 | -0.285 | -0.231 | -0.317 | -0.261 | -0.614 | 0.132 | 0.631 |
|  | KURTOSIS | 3.246 | 3.281 | 2.543 | 2.846 | 2.893 | 3.010 | 2.777 | 2.740 | 2.800 | 3.485 | 2.743 | 2.323 |
| FOLK AND | MEAN | 18.27 | 17.56 | 32.32 | 63.93 | 17.79 | 26.00 | 18.48 | 18.26 | 18.41 | 17.98 | 32.19 | 52.18 |
| WARD METHOD | SORTING | 3.065 | 3.022 | 2.734 | 2.324 | 3.041 | 2.341 | 3.045 | 3.045 | 3.031 | 3.001 | 2.728 | 2.325 |
| (mm) | SKEWNESS | 0.203 | 0.197 | 0.612 | -0.939 | 0.198 | 0.605 | 0.208 | 0.204 | 0.206 | 0.200 | 0.612 | -0.378 |
|  | KURTOSIS | 15.88 | 1.160 | 0.646 | 0.430 | 1.152 | 0.944 | 0.938 | 0.943 | 0.939 | 17.67 | 0.903 | 0.428 |
| FOLK AND | MEAN | 5.775 | 5.831 | 4.951 | 3.967 | 5.813 | 5.265 | 5.758 | 5.775 | 5.763 | 5.798 | 4.957 | 4.260 |
| WARD METHOD | SORTING | 1.616 | 1.596 | 1.451 | 1.217 | 1.605 | 1.227 | 1.606 | 1.607 | 1.600 | 1.586 | 1.448 | 1.217 |
| (f) | SKEWNESS | -0.203 | -0.197 | -0.612 | 0.939 | -0.198 | -0.605 | -0.208 | -0.204 | -0.206 | -0.200 | -0.612 | 0.378 |
|  | KURTOSIS | 15.88 | 1.160 | 0.646 | 0.430 | 1.152 | 0.944 | 0.938 | 0.943 | 0.939 | 17.67 | 0.903 | 0.428 |
| FOLK AND | MEAN: | Coarse Silt | Coarse Silt | Very Coarse Silt | Very Fine Sand | Coarse Silt | Coarse Silt | Coarse Silt | Coarse Silt | Coarse Silt | Coarse Silt | Very Coarse Silt | Very Coarse Silt |
| WARD METHOD | SORTING: | Poorly Sorted | Poorly Sorted | Poorly Sorted | Poorly Sorted | Poorly Sorted | Poorly Sorted | Poorly Sorted | Poorly Sorted | Poorly Sorted | Poorly Sorted | Poorly Sorted | Poorly Sorted |
| (Description) | SKEWNESS: | Coarse Skewed | Coarse Skewed | Very Coarse Skewed | Very Fine Skewed | Coarse Skewed | Very Coarse Skewed | Coarse Skewed | Coarse Skewed | Coarse Skewed | Coarse Skewed | Very Coarse Skewed | Very Fine Skewed |
|  | KURTOSIS: | Extremely Leptokurtic | Leptokurtic | Very Platykurtic | Very Platykurtic | Leptokurtic | Mesokurtic | Mesokurtic | Mesokurtic | Mesokurtic | Extremely Leptokurtic | Mesokurtic | Very Platykurtic |

|  | **SAMPLE STATISTICS** | | |  |  |  |  |  |  |  |  |  |
| --- | --- | --- | --- | --- | --- | --- | --- | --- | --- | --- | --- | --- |
|  |  | **220** | **240** | **260** | **275** | **308** | **340** | **357** | **390** | **420** | **440** | **460** |
|  | SAMPLE TYPE: | Polymodal, Poorly Sorted | Polymodal, Poorly Sorted | Polymodal, Poorly Sorted | Trimodal, Poorly Sorted | Unimodal, Moderately Well Sorted | Unimodal, Moderately Sorted | Unimodal, Moderately Sorted | Trimodal, Poorly Sorted | Trimodal, Poorly Sorted | Trimodal, Poorly Sorted | Polymodal, Poorly Sorted |
|  | TEXTURAL GROUP: | Sandy Mud | Sandy Mud | Sandy Mud | Sandy Mud | Sand | Muddy Sand | Muddy Sand | Sandy Mud | Sandy Mud | Sandy Mud | Sandy Mud |
|  | SEDIMENT NAME: | Very Fine Sandy Coarse Silt | Very Fine Sandy Coarse Silt | Very Fine Sandy Coarse Silt | Very Fine Sandy Coarse Silt | Moderately Well Sorted Fine Sand | Coarse Silty Fine Sand | Coarse Silty Fine Sand | Very Fine Sandy Coarse Silt | Very Fine Sandy Coarse Silt | Very Fine Sandy Coarse Silt | Very Fine Sandy Coarse Silt |
| METHOD OF | MEAN | 35.64 | 23.82 | 28.78 | 25.12 | 110.7 | 108.7 | 110.7 | 26.60 | 27.34 | 32.07 | 32.84 |
| MOMENTS | SORTING | 39.60 | 33.09 | 35.37 | 32.28 | 36.00 | 38.06 | 36.86 | 31.47 | 31.76 | 35.43 | 37.53 |
| Arithmetic (mm) | SKEWNESS | 1.526 | 2.375 | 2.038 | 2.332 | -1.890 | -1.711 | -1.910 | 2.071 | 2.035 | 1.754 | 1.694 |
|  | KURTOSIS | 3.858 | 7.452 | 5.819 | 7.387 | 5.096 | 4.350 | 5.072 | 6.513 | 6.328 | 4.908 | 4.517 |
| METHOD OF | MEAN | 21.30 | 13.28 | 17.23 | 14.50 | 95.79 | 92.62 | 94.78 | 16.06 | 16.72 | 19.94 | 19.62 |
| MOMENTS | SORTING | 2.703 | 2.680 | 2.599 | 2.790 | 2.060 | 2.097 | 2.113 | 2.628 | 2.603 | 2.585 | 2.682 |
| Geometric (mm) | SKEWNESS | 0.280 | 0.745 | 0.474 | 0.069 | -3.131 | -2.662 | -2.979 | 0.378 | 0.340 | 0.265 | 0.317 |
|  | KURTOSIS | 2.902 | 3.272 | 3.330 | 3.672 | 13.82 | 10.36 | 12.55 | 2.955 | 3.023 | 3.143 | 2.973 |
| METHOD OF | MEAN | 5.553 | 6.235 | 5.859 | 6.107 | 3.384 | 3.433 | 3.399 | 5.960 | 5.903 | 5.648 | 5.671 |
| MOMENTS | SORTING | 1.435 | 1.422 | 1.378 | 1.480 | 1.043 | 1.068 | 1.079 | 1.394 | 1.380 | 1.370 | 1.423 |
| Logarithmic (f) | SKEWNESS | -0.280 | -0.745 | -0.474 | -0.069 | 3.131 | 2.662 | 2.979 | -0.378 | -0.340 | -0.265 | -0.317 |
|  | KURTOSIS | 2.902 | 3.272 | 3.330 | 3.672 | 13.82 | 10.36 | 12.55 | 2.955 | 3.023 | 3.143 | 2.973 |
| FOLK AND | MEAN | 26.02 | 11.32 | 17.96 | 11.42 | 103.6 | 103.0 | 103.7 | 17.85 | 17.92 | 18.32 | 18.28 |
| WARD METHOD | SORTING | 2.345 | 2.175 | 3.016 | 2.171 | 1.624 | 1.632 | 1.625 | 3.013 | 3.012 | 3.011 | 3.030 |
| (mm) | SKEWNESS | 0.604 | -0.295 | 0.199 | -0.312 | -0.916 | -0.915 | -0.917 | 0.195 | 0.195 | 0.204 | 0.204 |
|  | KURTOSIS | 0.942 | 1.154 | 16.62 | 1.172 | 14.75 | 14.46 | 14.95 | 1.159 | 1.168 | 16.73 | 16.22 |
| FOLK AND | MEAN | 5.264 | 6.466 | 5.799 | 6.453 | 3.270 | 3.279 | 3.269 | 5.808 | 5.802 | 5.770 | 5.774 |
| WARD METHOD | SORTING | 1.230 | 1.121 | 1.593 | 1.119 | 0.700 | 0.707 | 0.701 | 1.591 | 1.591 | 1.590 | 1.599 |
| (f) | SKEWNESS | -0.604 | 0.295 | -0.199 | 0.312 | 0.916 | 0.915 | 0.917 | -0.195 | -0.195 | -0.204 | -0.204 |
|  | KURTOSIS | 0.942 | 1.154 | 16.62 | 1.172 | 14.75 | 14.46 | 14.95 | 1.159 | 1.168 | 16.73 | 16.22 |
| FOLK AND | MEAN: | Coarse Silt | Medium Silt | Coarse Silt | Medium Silt | Very Fine Sand | Very Fine Sand | Very Fine Sand | Coarse Silt | Coarse Silt | Coarse Silt | Coarse Silt |
| WARD METHOD | SORTING: | Poorly Sorted | Poorly Sorted | Poorly Sorted | Poorly Sorted | Moderately Well Sorted | Moderately Sorted | Moderately Sorted | Poorly Sorted | Poorly Sorted | Poorly Sorted | Poorly Sorted |
| (Description) | SKEWNESS: | Very Coarse Skewed | Fine Skewed | Coarse Skewed | Very Fine Skewed | Very Fine Skewed | Very Fine Skewed | Very Fine Skewed | Coarse Skewed | Coarse Skewed | Coarse Skewed | Coarse Skewed |
|  | KURTOSIS: | Mesokurtic | Leptokurtic | Extremely Leptokurtic | Leptokurtic | Extremely Leptokurtic | Extremely Leptokurtic | Extremely Leptokurtic | Leptokurtic | Leptokurtic | Extremely Leptokurtic | Extremely Leptokurtic |

# SI4. BC granulometry: grain size distribution and statistic results.

|  | **SAMPLE STATISTICS** | | |  |  |  |  |  |  |  |  |  |  |
| --- | --- | --- | --- | --- | --- | --- | --- | --- | --- | --- | --- | --- | --- |
|  |  | **5** | **30** | **43** | **116** | **134** | **145** | **249** | **251** | **270** | **301** | **314** | **360** |
|  | SAMPLE TYPE: | Bimodal, Very Poorly Sorted | Polymodal, Very Poorly Sorted | Polymodal, Very Poorly Sorted | Polymodal, Very Poorly Sorted | Bimodal, Very Poorly Sorted | Trimodal, Very Poorly Sorted | Polymodal, Very Poorly Sorted | Unimodal, Moderately Well Sorted | Unimodal, Moderately Well Sorted | Unimodal, Well Sorted | Unimodal, Poorly Sorted | Unimodal, Moderately Well Sorted |
|  | TEXTURAL GROUP: | Sandy Mud | Mud | Sandy Mud | Sandy Mud | Sandy Mud | Sandy Mud | Mud | Muddy Sand | Sand | Sand | Muddy Sand | Muddy Sand |
|  | SEDIMENT NAME: | Very Fine Sandy Very Coarse Silt | Medium Silt | Very Fine Sandy Very Coarse Silt | Very Fine Sandy Very Coarse Silt | Very Fine Sandy Very Coarse Silt | Very Fine Sandy Very Coarse Silt | Fine Silt | Very Coarse Silty Very Fine Sand | Moderately Well Sorted Very Fine Sand | Well Sorted Very Fine Sand | Very Coarse Silty Very Fine Sand | Very Coarse Silty Fine Sand |
| METHOD OF | MEAN | 29.67 | 21.37 | 27.91 | 47.60 | 100.5 | 34.61 | 17.85 | 112.5 | 116.1 | 113.5 | 69.75 | 120.6 |
| MOMENTS | SORTING | 31.46 | 39.73 | 42.65 | 97.83 | 197.2 | 45.18 | 40.53 | 41.88 | 43.74 | 35.14 | 36.78 | 49.26 |
| Arithmetic (μm) | SKEWNESS | 2.067 | 4.098 | 3.135 | 3.808 | 3.083 | 3.622 | 3.760 | 0.043 | -0.231 | -0.175 | 0.336 | -0.094 |
|  | KURTOSIS | 9.906 | 22.66 | 15.02 | 18.25 | 11.38 | 23.75 | 18.10 | 3.316 | 3.519 | 4.095 | 3.475 | 2.916 |
| METHOD OF | MEAN | 14.26 | 7.599 | 9.996 | 13.69 | 32.49 | 13.99 | 4.948 | 99.20 | 99.49 | 102.7 | 51.79 | 101.9 |
| MOMENTS | SORTING | 4.223 | 4.375 | 4.860 | 5.281 | 4.913 | 4.919 | 4.320 | 1.978 | 2.184 | 1.895 | 2.912 | 2.232 |
| Geometric (μm) | SKEWNESS | -0.617 | 0.090 | -0.135 | -0.054 | -0.376 | -0.466 | 0.701 | -4.368 | -3.931 | -5.357 | -2.482 | -3.724 |
|  | KURTOSIS | 2.365 | 2.324 | 2.027 | 2.429 | 3.477 | 2.052 | 3.012 | 28.97 | 21.79 | 38.40 | 9.331 | 20.54 |
| METHOD OF | MEAN | 6.132 | 7.040 | 6.644 | 6.191 | 4.944 | 6.159 | 7.659 | 3.334 | 3.329 | 3.284 | 4.271 | 3.295 |
| MOMENTS | SORTING | 2.078 | 2.129 | 2.281 | 2.401 | 2.296 | 2.298 | 2.111 | 0.984 | 1.127 | 0.922 | 1.542 | 1.159 |
| Logarithmic (φ) | SKEWNESS | 0.617 | -0.090 | 0.135 | 0.054 | 0.376 | 0.466 | -0.701 | 4.368 | 3.931 | 5.357 | 2.482 | 3.724 |
|  | KURTOSIS | 2.365 | 2.324 | 2.027 | 2.429 | 3.477 | 2.052 | 3.012 | 28.97 | 21.79 | 38.40 | 9.331 | 20.54 |
| FOLK AND | MEAN | 14.23 | 7.200 | 9.497 | 12.55 | 29.55 | 14.15 | 4.629 | 108.4 | 113.3 | 111.1 | 63.28 | 114.7 |
| WARD METHOD | SORTING | 4.378 | 4.570 | 5.097 | 5.485 | 5.069 | 4.932 | 4.478 | 1.500 | 1.544 | 1.339 | 2.275 | 1.607 |
| (μm) | SKEWNESS | -0.393 | -0.048 | -0.136 | -0.211 | -0.224 | -0.474 | 0.193 | -0.204 | -0.277 | -0.077 | -0.443 | -0.283 |
|  | KURTOSIS | 0.871 | 0.841 | 0.790 | 0.976 | 1.953 | 0.757 | 1.114 | 1.334 | 1.673 | 1.092 | 2.242 | 1.325 |
| FOLK AND | MEAN | 6.135 | 7.118 | 6.718 | 6.316 | 5.081 | 6.143 | 7.755 | 3.205 | 3.142 | 3.170 | 3.982 | 3.123 |
| WARD METHOD | SORTING | 2.130 | 2.192 | 2.350 | 2.456 | 2.342 | 2.302 | 2.163 | 0.585 | 0.627 | 0.421 | 1.186 | 0.684 |
| (φ) | SKEWNESS | 0.393 | 0.048 | 0.136 | 0.211 | 0.224 | 0.474 | -0.193 | 0.204 | 0.277 | 0.077 | 0.443 | 0.283 |
|  | KURTOSIS | 0.871 | 0.841 | 0.790 | 0.976 | 1.953 | 0.757 | 1.114 | 1.334 | 1.673 | 1.092 | 2.242 | 1.325 |
| FOLK AND | MEAN: | Medium Silt | Fine Silt | Medium Silt | Medium Silt | Coarse Silt | Medium Silt | Fine Silt | Very Fine Sand | Very Fine Sand | Very Fine Sand | Very Fine Sand | Very Fine Sand |
| WARD METHOD | SORTING: | Very Poorly Sorted | Very Poorly Sorted | Very Poorly Sorted | Very Poorly Sorted | Very Poorly Sorted | Very Poorly Sorted | Very Poorly Sorted | Moderately Well Sorted | Moderately Well Sorted | Well Sorted | Poorly Sorted | Moderately Well Sorted |
| (Description) | SKEWNESS: | Very Fine Skewed | Symmetrical | Fine Skewed | Fine Skewed | Fine Skewed | Very Fine Skewed | Coarse Skewed | Fine Skewed | Fine Skewed | Symmetrical | Very Fine Skewed | Fine Skewed |
|  | KURTOSIS: | Platykurtic | Platykurtic | Platykurtic | Mesokurtic | Very Leptokurtic | Platykurtic | Leptokurtic | Leptokurtic | Very Leptokurtic | Mesokurtic | Very Leptokurtic | Leptokurtic |
